# Self-regulation of effort for a better health-related quality of life: a multidimensional activity pacing model for chronic pain and fatigue management

**DOI:** 10.1080/07853890.2023.2270688

**Published:** 2023-10-23

**Authors:** Ioulia Barakou, Katie L. Hackett, Tracy Finch, Florentina Johanna Hettinga

**Affiliations:** a Department of Nursing, Midwifery & Health, Northumbria University, Newcastle upon Tyne, UK; b Department of Social Work, Education and Community Wellbeing, Northumbria University, Newcastle upon Tyne, UK; c CRESTA Fatigue Clinic, Newcastle upon Tyne Hospitals NHS Foundation Trust, Newcastle upon Tyne, UK; d Department of Sport Exercise and Rehabilitation, Northumbria University, Newcastle upon Tyne, UK

**Keywords:** Chronic conditions, physical activity behaviour, pain, fatigue management, self-regulation, quality of life

## Abstract

**Purpose:**

To propose a comprehensive multidimensional model of activity pacing that improves health-related quality of life and promotes sustained physical activity engagement among adults with chronic conditions.

**Materials and methods:**

A narrative review was conducted to examine the existing literature on activity pacing, health-related quality of life, pain and fatigue management, and physical activity promotion in chronic conditions.

**Results:**

The literature revealed a lack of a cohesive approach towards a multidimensional model for using activity pacing to improve health-related quality of life. A comprehensive multidimensional model of activity pacing was proposed, emphasizing the importance of considering all aspects of pacing for sustained physical activity engagement and improved health-related quality of life. The model incorporates elements such as rest breaks, self-regulatory skills, environmental factors, and effective coping strategies for depression/anxiety. It takes into account physical, psychological, and environmental factors, all of which contribute significantly to the enhancement of health-related quality of life, physical function, and overall well-being, reflecting a holistic approach.

**Conclusions:**

The model offers guidance to researchers and clinicians in effectively educating patients on activity pacing acquisition and in developing effective interventions to enhance physical activity engagement and health outcomes among adults with chronic conditions. Additionally, it serves as a tool towards facilitating discussions on sustained physical activity and a healthy lifestyle for patients, which can eventually lead to improved quality of life.

## 1. Introduction

Among adults with chronic conditions (e.g. chronic pain conditions, cardiovascular diseases, neurological conditions, arthritis, cancer, and gastrointestinal conditions), consistent engagement in physical activity is a significant element of a healthy lifestyle [1–3]. Sustained participation in physical activity has been shown to alleviate pain and fatigue symptoms and improve health-related quality of life (HRQoL) in these individuals [4–13]. Despite these benefits, adults with chronic conditions often experience significant symptoms of pain and fatigue associated with their medical illness [14,15], which can act as barriers to physical activity participation. Fear avoidance behaviour, where individuals avoid physical activities in anticipation of pain and fatigue is a common activity engagement barrier [16–21], which may lead to lower physical activity levels in adults with chronic conditions [21,22], compared to those without [23]. Additionally, overactivity is also problematic in adults who experience fatigue as some people push beyond their limitations, which can contribute to a boom-and-bust cycle of activity leading to prolonged periods of rest [16,24]. Therefore, both underactivity and overactivity are associated with disability [24]. Thus, it is essential to explore strategies to support sustained physical activity behaviours as a physical activity offers various health benefits, including improved mental health, dementia, and sleep as well as alleviation of fatigue symptoms [25,26]. Activity pacing has been identified as a promising solution to overcome these barriers, as it aims to increase individuals’ engagement in activities and manage pain and fatigue adequately in adults with chronic conditions [27–29].

Activity pacing, as a fatigue and pain management strategy, involves the regulation of energy and activity levels in adults with chronic conditions, with the goal of maintaining or increasing engagement in activities and encompasses pacing periods of rest [16,21,30–32]. It was first introduced in healthcare settings in 1976 as part of the conceptualization of operant principles in chronic pain, a system for promoting exercise [33]. Fordyce recognised that individuals with chronic conditions often struggled with activity pacing, resulting in a recurring pattern of limited exercise and activity followed by overexertion and subsequent pain (contingency periods), which often led to the avoidance of physical activity and exercise [33,34]. Fordyce developed an operant program to overcome these issues highlighting the importance of planned activities and rest during the day, gradually and systematically planned, instead of contingency periods in adults with chronic conditions. The energy conservation approach was also developed emphasising the energy expended by the patient, aiming to achieve a balance between fulfilling daily meaningful tasks and taking necessary rest to alleviate pain and fatigue [30]. Nevertheless, results of activity pacing interventions were found to be promising but inconclusive [29] and in recent study, operant learning has been found to be more advantageous compared to the energy conservation approach [35]. Later, the contemporary interest in pacing in healthcare settings shifted to advocating tailored approaches to fatigue management [26,36,37], while a prospective cohort study among survivors of cardiac arrest described planning, pacing, doing activities, and simplifying activities as fatigue-related problems [38]. Yet, the concept of pacing had already sparked numerous discussions within the field of sport and exercise sciences [39].

Intriguingly, the regulation of effort and energy expenditure has been systematically investigated in sport and exercise science since the beginning of this century [40,41], particularly in endurance sports, where athletes strive to achieve optimal performance by finishing first or with the shortest possible time [16,39,42,43]. In contrast, the goal in healthcare settings is for individuals to manage to do everything they want during the day [16]. Despite the differences in goals between sports and healthcare settings, Abonie et al. [16] argued that the underlying mechanism of pacing in sports is similar to activity pacing in healthcare contexts, as the mechanism related to the regulation of energy levels can be applied to daily activity pacing for individuals with chronic conditions. Therefore, the insights gained from sport and exercise sciences such as the multidimensionality and the importance of incorporating the environment [43] can inform activity pacing interventions, where the exploration of underpinning mechanisms has been limited [16]. Consequently, activity pacing can be considered a lifestyle strategy [16,44]. More specifically, in healthcare settings, activity pacing has been briefly described as a multidimensional concept with researchers recognising the complexity of pacing [43,45–47]; However, limited evidence is available, which might limit the optimal application of activity pacing in healthcare settings and in research. Therefore, a more comprehensive discussion about activity pacing dimensionality is needed to guide health professionals and researchers in positively impacting pain and fatigue management.

In addition to its potential impact on pain and fatigue management, activity pacing may also have a significant role in promoting exercise engagement. Activity pacing is a proposed strategy to manage exercise intensity, potentially enhancing the enjoyment of endurance. Empirical evidence suggests exercise enjoyment decreases as intensity increases [48–51], with moderate intensity activities being more enjoyable for some individuals [51]. Exercise enjoyment, in turn, positively predicts exercise habit frequency, and intention to continue being active [52]. Moreover, moderate exercise has also been linked to future exercise behaviour, indicating that those who enjoy exercise are more likely to maintain their engagement in physical activity in the future [51]. A study among patients with heart failure has identified that motivation and physical activity enjoyment were positively correlated [53], while another study recognised physical activity enjoyment as an important motivational factor [54]. Helping individuals with chronic conditions to learn how to regulate their effort through activity pacing, can contribute to improved physical and psychological outcomes. Behaviour change techniques such as cognitive behaviour therapy might also be effective in promoting physical activity throughout the day [55,56]. Consequently, highlighting the importance managing exercise intensity through activity pacing may play a leading role in facilitating sustained physical activity engagement as well as inform the development of tailored interventions.

Therefore, this narrative review focuses on activity pacing as a solution to overcome the challenges people with chronic conditions experience and how it can support long term and sustained engagement in physical activity, as well as exploring its dimensionality, benefits, and application in research. This review aims to develop a new multidimensional model of activity pacing, incorporating theories on activity pacing, self-regulation, and multidimensionality of fatigue and energy regulation and provides practical guidance for practitioners. Based on all the above, the specific aims of this review are [1]: to explore the dimensionality of activity pacing and [2] to develop a comprehensive and practically oriented multidimensional model of activity pacing based on a theories-informed approach that can be applied in health and care research.

By synthesizing existing empirical and conceptual literature on activity pacing, this narrative review brings together novel insights into the activity pacing concept, which will facilitate discussions on enhancing sustained physical activity and HRQoL and reducing pain and fatigue among individuals with chronic conditions. The proposed model can serve as a useful tool for researchers and practitioners alike, facilitating discussions with patients about how to integrate physical activity in their daily lives, and guiding the development of new interventions aimed at promoting activity pacing in the context of chronic conditions.

## 2. Search strategy

In this narrative review, we investigated the main topic of ‘activity pacing’ by conducting searches across four electronic databases: Web of Science, Medline (ProQuest), EbscoHost, and Scopus, in September 2023. All abstracts retained were reviewed to ensure their alignment with the review’s focus. Moreover, additional relevant articles were incorporated based on expert knowledge, screening of reference lists of relevant articles, and to supplement specific literature for sub-topics, enriching the scope and depth of this review. Only articles and/or reviews published in English were included.

## 3. Exploration of activity pacing in research

The activity pacing concept has been introduced as a promising solution to pain and fatigue management in adults with chronic conditions. In the healthcare context, in 1984, Wiener described activity pacing as fluctuating based on the monitoring of the physiological imperative to manage symptoms in adults with rheumatoid arthritis [57]. Since then, numerous studies have applied and researched activity pacing as a concept in which individuals break down activities into smaller, more manageable tasks through self-regulation to manage symptoms of pain and fatigue and maintain physical activity engagement across various chronic conditions [31,32,58–63]. However, there is a lack of clarity regarding whether individuals pace their activities due to symptom contingency or in anticipation of fatigue, as came forward from a recent meta-analysis [64]. Symptom contingency refers to the behaviour of increasing pacing as a response to experiencing high symptoms of pain and fatigue [16,21,64], while anticipation of fatigue refers to pacing activities to balance exercise regulation more evenly, yet this could also be done by potentially completely avoiding physical activity to prevent the occurrence of imminent symptoms [36,65]. As a result, activity pacing interventions are needed to help individuals develop pacing patterns of activities and rest, tailored to whether they employ symptom contingency or anticipation of fatigue approaches [36]. Therefore, recently, activity pacing research has been conducted to further explore and understand activity pacing behaviours among adults with chronic conditions, both conceptually and empirically.

### 3.1. Conceptual literature

Qualitative studies significantly enhance our understanding of activity pacing from various perspectives, as they offer researchers an opportunity to delve into patients’ viewpoints [66]. Particularly, the literature emphasizes co-creation as a valuable healthcare approach, which fosters active involvement of stakeholders and healthcare professionals in public health services, thereby allowing for a tailored approach that meets individuals’ needs [67–70]. Involving patients in research through interviews or focus groups serves as a means to incorporate their insights and experiences [66]. A qualitative study explored the views and beliefs about activity pacing among adults with long-term conditions (chronic low back pain, chronic widespread pain, fibromyalgia and chronic fatigue syndrome) [60]. The study revealed that activity pacing is perceived as a multifaceted strategy that involves activities, exercise, breaks, rest, social events, social environment, and self-regulation. Participants also reported that activity pacing helped them to cope with pain and fatigue symptoms [60], thus acknowledging the multidimensional nature of the concept. In another study, participants discussed how activity pacing helped them with housework, employment, family, and leisure activities and was perceived as a beneficial strategy for managing pain symptoms [71]. Similarly, in another qualitative study, activity pacing was reported to be a beneficial strategy for managing fatigue symptoms while the participants discussed various factors that facilitated their pacing behaviour [72]. Specifically, environmental and social factors were identified as important aspects for coping with pain symptoms, highlighting the influences of external context on activity pacing behaviour [72]. In addition, adults with rheumatic disease lacked knowledge on fatigue, which could pose a challenge to managing their symptoms as fatigue is a multidimensional concept that encompasses physical and psychological factors [73]. Despite reporting positive reviews of activity pacing, participants still face challenges related to fatigue in their everyday lives. The researchers suggested that social support could be helpful for these individuals in fatigue management [73]. Additionally, setting goals and prioritising activities were also found to be essential factors for coping with fatigue in individuals with rheumatic disease. Another recent interview study explored the perceptions and experiences of stroke survivors on activity pacing behaviour, with participants reporting the need for instructions on activity pacing to better manage fatigue and remain engaged in physical activity [74]. Overall, individuals with chronic conditions perceive activity pacing as a concept with various dimensions that contribute to pain and fatigue management.

In a similar vein, the active involvement of healthcare professionals in research co-creation research is also important [75], encompassing valuable insights, information and promoting collaboration [70,76]. Therefore, the collaboration between researchers, healthcare professionals and stakeholders could result in service improvement [66]. A qualitative study among physiotherapists highlighted the significance of personal goals in activity pacing, considering both cognitive and physical experiences equally important [77]. The researchers concluded that activity pacing encompasses various aspects, including self-efficacy, cognitive behaviour, experiential learning, and stages of change [77]. Hence, it is evident that both individuals experiencing pain and fatigue and healthcare professionals perceive activity pacing as a concept with diverse dimensions, all of which contribute to pain and fatigue management. Nevertheless, rigorous qualitative research is recommended in a range of populations with chronic conditions to comprehensively capture the holistic view of activity pacing concept as a current limitation is that only a selection of chronic conditions has been interviewed.

### 3.2. Empirical literature

Despite these indications of multidimensionality reported by various chronic condition populations, activity pacing research has primarily focused on pain, fatigue, and physical activity, without considering any moderating factors. While promising results have been reported for pain and fatigue management, the omission of dimensions impacting on activity pacing, such as social support, cognitive factors, and rest, suggests exploration is needed. A study on adults with osteoarthritis found that tailored activity pacing approach may be effective in managing fatigue [78], while on another study among adults with multiple sclerosis effectively improved physical activity levels [29]. Antcliff’s framework, which aims to improve patients’ function and quality of life, was integrated into clinical settings for adults with various chronic conditions who experience chronic pain and fatigue, and it was the first to consider different activity pacing dimensions. The next step would now be to also account for environmental and social support aspects [44,47]. This intervention study found that adults with chronic pain and fatigue reported improvements in pain, fatigue, quality of life, and psychological well-being [47]. Although promising, most intervention studies do not consider the multidimensionality of the activity pacing concept. Thus, to be able to offer a tailored multidimensional approach to activity pacing, the use of an evidence-based approach involving available theories and stakeholders’ perceptions is important to develop an underpinning rationale on which pacing factors to consider in the model.

## 4. Activity pacing as a dynamic multidimensional concept

The operant learning theory by Fordyce was the first to emphasize the importance of optimally managing activities and rest to reduce chronic pain in adults with chronic conditions [33]. Although the operant learning theory has had a positive impact on chronic pain research and rehabilitation, it is argued that activity pacing is not a treatment for chronic pain, but it constitutes only one component of a multidisciplinary concept [45,46]. In the sport and exercise settings, the pacing concept was first discussed and recognised as a multidimensional concept by Smits et al. [43] highlighting human environment interactions and broadening applications in exercise regulation [43]. This was further applied to sports settings by Hettinga et al. [79] and Konings et al. [80]. In healthcare settings, over the years, there is some discussion over activity pacing’s dimensionality [81]. Kindermans et al. conducted a factor analysis on different pacing subscale items of different questionnaires (Chronic Pain Coping Inventory, Pain and Activity Relations Questionnaires, Patterns of Activity Measure-Pain) and found that activity pacing is unidimensional [81]. However, research undertaken in the context of rehabilitation recognised activity pacing as a multidimensional concept due to the involvement of different facets [47]. Antcliff and colleagues developed an activity pacing framework based on healthcare practice as a rehabilitative strategy [47], which includes the values of quota contingency and the operant approach aiming to help with the avoidance of activity by setting goals [47]. The framework aims to improve physical and cognitive function among individuals with chronic pain and fatigue while there is regular participation in physical activity and while symptoms of pain and fatigue are regulated. The operant approach has been previously found to encourage reductions in activity avoidance among patients with fibromyalgia syndrome [35]. Moreover, a systematic review/meta-analysis on activity pacing by Casson et al. [82] concluded that activity pacing interventions effectively alleviate fatigue symptoms, psychological distress, and depression while improving physical function in adults with chronic fatigue syndrome. Another study on the obstacles of activity pacing among adults with chronic pain noted that several obstacles limit activity pacing including memory and attention issues, as many people seemed to forget to take breaks [83].

Overall, this indicates a movement towards multidimensionality of activity pacing in the healthcare settings, with a growing emphasis on identifying and accounting for various factors that can impact its effectiveness. Despite this, the role of the environment as a key factor influencing the activity pacing strategy of individuals with chronic conditions has not yet been explored in the healthcare literature. Notably, a recent review by Sakalidis et al. has highlighted realistic goal setting as an important self-regulatory factor of sports participation and performance that keeps people motivated and engaged in the learning process [44]. The authors further focused on self-regulation and social environment in sports [44,84] explaining the importance of other exercisers and coaches on the athlete’s pacing strategy. It was emphasized that the social environment may be an important facilitator of self-regulation and athletic performance and pacing. In healthcare, the lack of attention to environmental factors as key aspects for activity pacing represents a notable gap in both theories and practical applications. As such, it is important to draw upon the insights and frameworks from sport and exercise science in underpinning activity pacing models in healthcare settings.

### 4.1. Breaking down multidimensionality

Based on preceding qualitative and quantitative evidence, activity pacing can be identified as a multidimensional concept that encompasses the regulation of effort and management of pain and fatigue. Abonie suggested that without training or intervention, there is not a clear pacing strategy that helps with the improvement of the HRQoL amongst people with multiple sclerosis [36]. Multiple factors could affect activity pacing, which calls for a holistic approach that enhances chronic condition management, HRQoL, physical functioning, and overall well-being. Relatedly, Antcliff’s activity pacing framework highlights the need for improving patients’ function and quality of life rather than solely focusing on pain and fatigue management [47]. Chronic conditions negatively affect HRQoL and physical function [85,86]. Broadly, it is acknowledged that HRQoL is a multidimensional concept comprising physical, psychological, and social functioning [85] that are often affected by chronic conditions and their treatment. In addition, Wilson and Cleary’s conceptual model highlights the multidimensionality of HRQoL [86], which consists of physiological variables, symptoms, functionality, and health perceptions. The authors claim that each dimension is linked to and influences the following dimension (physiological variables → symptoms → functionality → health perceptions → HRQoL) [87]. Thus, activity pacing may contribute to better HRQoL, physical function, and well-being in adults with chronic conditions by managing physical, psychological, and social factors. Therefore, we propose a holistic model for building activity pacing interventions that improve HRQoL through promoting physical activity.

#### 4.1.1. Physical factors

Numerous health factors have been associated with HRQoL and well-being in chronic conditions. Among these factors, physical inactivity has been associated with impaired HRQoL in patients with asthma and diabetes [86,88]. Additionally, individuals with chronic fatigue syndrome have been found to commonly lack physical activity as noted in a review article [89]. Activity avoidance is also a common behaviour among individuals with chronic pain as such behaviour may be attributed to the need to conserve energy and prevent further depletion of energy costs [90]. This behaviour may stem from a lack of motivation towards physical activity or apprehension regarding the potential consequences of engaging in physical activity such as exacerbating pain and fatigue symptoms or experiencing a flare-up of other symptoms [91,92]. Additionally, multiple cross-sectional and longitudinal studies indicate that physical activity engagement is associated with better HRQoL [93–95]. Physical activity also is a central role to activity pacing, crucial for managing chronic conditions and optimizing overall health [16,28]. For example, a study of 137 fibromyalgia participants demonstrated that exercise could improve physical and psychological functioning [96]. Moreover, physical activity can positively impact depression and anxiety in adults with cardiovascular diseases [97]. Randomised controlled trials among healthy (older) adults demonstrated that exercise programs reduced stress, depressive symptoms, and anxiety levels but also improved how individuals perceive their health status compared to the control group [98,99]. In summary, interdisciplinary evidence supports physical activity’s positive impact on HRQoL, physical function, well-being, and happiness in adults and older adults [93,99–101]. Therefore, optimal physical activity participation is critical for individuals with chronic conditions.

However, physical activity engagement for adults with chronic conditions often faces several barriers. In a qualitative study interviewing individuals from four chronic disease populations (heart failure, stroke, diabetes, and chronic obstructive pulmonary disease), participants reported barriers, including lack of motivation, lack of confidence with exercise, lack of health care professional support, and severity of physical symptoms [102]. Healthcare professionals in the same study perceived more barriers to physical activity participation than patients, possible indicating inadequate programs, facilities, and support [102]. A UK-based study found that participants with chronic conditions exercised less than healthy adults [103], suggesting a need for targeted interventions. While the literature on activity pacing and physical activity is still scarce, an intervention study in adults with multiple sclerosis found that activity pacing is effective in improving physical activity levels without worsening fatigue symptoms [29], promising for distributing efforts effectively, enabling individuals to engage in more activities. Nevertheless, more research is needed to consider all factors in activity pacing process.

Rest is another key factor in potentially improving HRQoL in adults with chronic conditions and is part of an activity pacing strategy involving both pacing activities and well-timed rest [73]. Despite its potential importance, literature on rest in chronic conditions is limited. Rest is considered both a physical and psychological factor [104,105], and is occasionally researched in medicine as a physiological need linked to sleep [104,106]. In psychology, rest is often associated with relaxation [104]. An explorative nursing study from 1970 found that rest varies based on individual needs and may involve different activities [107]. Rest is essential for alleviating stress in the body, mind, and spirit [105,107,108]. While physical rest, primarily through sleep, is most frequently studied, mental and spiritual rest are less mentioned [105]. Moreover, an intervention study among healthy adults using rest-breaks showed significant improvement in all behavioural measurements [109]. Rest breaks also benefit workplace fatigue in the general population [110]. Another exploratory study among healthy adolescents found rest breaks are essential when there is sleep loss [111]. Overall, rest can take on various forms, such as sleep, relaxation, inactivity, and active restoration, which allows individuals to take a break from demanding daily life [106,111,112]. This highlights the distinction between health and performance, and may contribute to the psychosocial model of health in chronic illness management. Rest should be considered a basis for physical health support [104] and an integral aspect of activity pacing approach, assisting healthcare professionals provide a holistic approach to managing chronic conditions [104]. Chronic conditions can take a physical and emotional toll on individuals, and proper rest might help individuals manage their symptoms, feel more energized and engage in daily activities. Moreover, appropriate rest is key to a successful activity pacing approach, yet literature primarily focuses on exercise/activity elements, lacking literature on the importance of rest in chronic conditions and activity pacing context. Further research should explore how adequate rest can improve HRQoL in activity pacing approaches for adults with chronic conditions.

Activity pacing and its impact on HRQoL and physical function are also influenced by pain and fatigue symptoms. Pain is prevalent in adults with chronic conditions and a leading cause of disability [113,114]. Extensive research has investigated pain in various chronic conditions, including cancer, multiple sclerosis, and musculoskeletal diseases [113–116]. Similarly, fatigue is common in adults with chronic illness and studied in conditions such as Parkinson’s disease, chronic obstructive pulmonary disease, rheumatoid arthritis, and inflammatory bowel disease [116–121]. A study exploring the effects of a tailored activity pacing intervention on pain and fatigue among adults with osteoarthritis found reduced reported fatigue but no effect on pain reduction compared to the control group [78]. Additionally, tailored activity pacing intervention has potential for alleviating fatigue and pain symptoms while allowing engagement in daily life activities and a healthy lifestyle [29]. In this context, regulating efforts adequately may contribute to pain and fatigue alleviation, but further research is necessary to better understand activity pacing effects.

Furthermore, the detrimental effects of pain and fatigue on HRQoL are extensively demonstrated. A study among adults with chronic pain found it was associated with impaired HRQoL [122]. Similarly, fatigue negatively impacts HRQoL in adults after stroke [123], with neurologic illnesses [124], and cancer [125]. More specifically, when people are fatigued, intense exercise can cause a negative affective load leading to strong fatigue responses, which then makes them less likely to engage in activities again [126,127]. Overall, it is established that pain and fatigue symptoms adversely impact HRQoL among adults with chronic conditions [128–131]. However, the classification of pain and fatigue as purely physical factors is debated. Both symptoms are complex, multidimensional experiences involving physical and psychological aspects [132,133]. Therefore, interventions targeting these symptoms require an individualised and holistic approach in chronic conditions.

#### 4.1.2. Psychological factors

As previously mentioned, pain and fatigue, both multidimensional constructs, significantly affect physical function in adults with chronic conditions and have been considered psychological variables in the literature [132–135]. A study among adults with various chronic conditions including fibromyalgia demonstrated that fatigue negatively influenced depression, pain intensity, and sleep disturbance [132]. The role of psychology in pain management highlights how pain could potentially lead to low affect and depression with avoidance of activity as a notable psychological trait of pain [135]. To effectively manage the psychological factors that impact physical function in adults with chronic conditions, such as pain and fatigue, the use of self-regulation skills is essential, particularly in the context of activity pacing. Self-regulation, a goal-directed behaviour or performance [136], involves an individual’s ability to engage in and follow strategies aimed at promoting positive emotional consciousness and expression and achieving personal goals or maintaining current standards [137]. It is also a key component of maintaining a balanced physically active lifestyle [138–140]. The four essential elements of self-regulation include goal setting, monitoring, control, and self-evaluation [136,141–143]. Additionally, self-regulation has been extensively studied in sports and exercise sciences with pacing behaviour viewed from a self-regulatory perspective. Zimmerman’s theory on self-regulation of learning includes pacing phases such as forethought, performance, and self-reflection, highlighting skills like planning, monitoring, and evaluating that could lead to optimal performance [144]. For instance, in sports context, athletes pre-plan race tactics and performance, monitor pacing behaviour during the race and evaluate actions and performance afterward [145]. In addition to its role in sports science, self-regulation is also a key part of health and health behaviour theories, recognised for its role in exercise regulation and potential to improve health and the effects of chronic illnesses [44,146]. For instance, a study among cancer patients evaluated a physical activity intervention emphasising self-regulatory skills in the active control group, resulting in sustained physical activity engagement [147]. In another intervention study with irritable bowel syndrome participants, two movement-based self-regulation strategies, walking, and yoga, were compared. Although both groups experienced benefits in symptoms and psychological well-being, the walking group had sustained symptom relief compared to the yoga group [148]. These findings suggest the potential impact of self-regulatory skills on physical activity and exercise adherence among people with chronic conditions. In fact, insufficient self-regulatory skills have been linked to low adherence to health-related exercise [149,150]. Studies on both young and older adults have also established the importance of self-regulation skills in promoting moderate to vigorous intensity physical activity [151,152]. Furthermore, the relationship between exercise intensity and enjoyment can have an impact on an individual’s psychological well-being. Learning to pace exercise intensity adequately may help prevent negative affective load and subsequently exercise avoidance [126]. Literature suggests that maintaining exercise intensity below the point of ventilation threshold can elicit a greater sense of pleasure, encouraging sustained exercise engagement [153]. Therefore, self-regulation of efforts is relevant not only for managing fatigue in people with long-term conditions and promoting sustained physical activity engagement but also for improving HRQoL [63,154–156].

Chronic conditions such as coronary heart disease, cancer, stroke, depression, and anxiety have been linked to impaired HRQoL [157–162]. Depression and anxiety are common mental health conditions that can hinder the effective management of chronic health condition [163–165]. More specifically, in multiple sclerosis, depression and anxiety are considered as highly debilitating symptoms that significantly impact individuals’ quality of life while pain and fatigue have also been associated with anxiety [166,167]. Fatigue can arise in interoceptive and sensorimotor systems; therefore, as fatigue levels increase, the perceived value of investing effort into a task diminishes, ultimately resulting in performance decrements [168]. Chalal et al. found a bi-directional relationship between fatigue and depression/anxiety [169]. Moreover, depression and anxiety can cause feelings of hopelessness, low energy, and difficulty concentrating, making it challenging to follow an activity pacing plan or engage in physical activity [170]. Furthermore, depression and anxiety can disrupt sleep, exacerbating fatigue management difficulties [171]. Given the limited literature on the role of depression and anxiety in activity pacing, it is important to acknowledge their impact and consider them in the activity pacing plan [47,82]. This may involve incorporating coping strategies, seeking therapy or considering medication to alleviate symptoms. Addressing depression and anxiety may improve symptom management, facilitate engagement in physical activity, and enhance adherence to activity pacing plans.

#### 4.1.3. Environmental factors

Psychosocial factors are important determinants of HRQoL in chronic conditions. A negative social environment is associated with a worsened HRQoL [159,172]. In addition, a lack of social support or pressures to overdo and overachieve health behaviours may challenge adherence to pacing plans [173,174]. An experimental study investigated verbal encouragement’s impact on exercise adherence among untrained but active individuals [175]. Results showed it positively influenced motivation to exercise and thereby sustained exercise adherence. The social environment’s role in pacing is discussed in the sport and exercise context, where athletes display different behaviours when racing alone versus against competitors [44]. Additionally, a recent study among athletes exploring training during COVID-19 lockdown, found that exercise motivation was reduced [176]. The authors highlighted this reduction to the absence of social facilitators and encouragement and reduced team interaction. Similarly, in healthcare settings, adequate social support and encouragement from friends and family can help individuals with chronic conditions stay motivated and consistent with pacing strategies and goals, impacting their physical and mental well-being [173, 174]. In addition to the social environment, the built environment including green spaces, streets, and leisure infrastructure [177], significantly impacts physical activity management [178–180] while a deeper understanding of this impact by health professionals and individuals with chronic conditions could positively affect HRQoL. When designed effectively, it promotes physical activity and enhances mental health, improving well-being [177,181,182]. In a 2020 study among healthy adults, the built environment affected the quality of life linked to physical and psychological factors [183]. However, the role of the social and built environment in activity pacing context has not been explored in previous literature. Further research is required to identify specific ways in which social and built environments influence activity pacing and physical activity in individuals with chronic conditions and to develop effective strategies for addressing challenges or barriers related to these factors. This knowledge will help healthcare providers and other professionals develop targeted and effective interventions to support individuals with chronic conditions, including the use of activity pacing as a key strategy.

## 5. Putting it all together: a multidimensional model of activity pacing

### 5.1. Summary of underpinning theories leading to the model

This review proposes a multidimensional model for activity pacing designed for use by researchers and healthcare professionals that incorporates a multidimensional approach to improve HRQoL, physical function, and well-being. The model is based on key theories and concepts of activity pacing, HRQoL, self-regulation, environment, and pain and fatigue management derived from a comprehensive review [16,29,33,44,47,63,78,86–87,146,184]. These underpinning theories guide the development of this model, aiding researchers and clinicians in a more effective approach to educating patients on activity pacing acquisition and will facilitate discussions toward a holistic tailored activity pacing intervention. In the context of sport and exercise science, pacing has been extensively discussed [42,43,185] and theoretical insights and models have been developed and tested experimentally [186], with self-regulation identified as an important factor for adequate pace regulation and long-term athletic excellence [184,187]. Within healthcare settings, activity pacing research has taken a more applied approach involving stakeholders and evaluating interventions, but with less focus on the development of the theoretical underpinnings relevant to activity pacing [78]. Abonie et al. [16] was the first to utilize pacing theories from sport and exercise science in developing a tailored pacing intervention [16,29]. Antcliff’s framework, based on stakeholders’ input, provides a more comprehensive approach based on practical applications highlighting the importance of the factors including physical activity, rest, pain/fatigue, self-regulation, and depression/anxiety [47]. The next step, based on recent theory, to include the impacts of environmental factors. More recently, Sakalidis’ model [44] highlighted the social environment’s role in sports participation, which has only been generally explored. Therefore, the proposed multidimensional model considers various physical, psychological, and environmental factors to enhance HRQoL in individuals with chronic conditions. Overall, the model uses a behaviour change approach to encourage individuals with chronic conditions to adopt lifestyle changes suitable for them, thereby promoting health-relating quality of life [188].

### 5.2. Activity pacing model

This narrative review utilizes the activity pacing theory, literature including stakeholders’ views and perceptions, and promising results of existing activity pacing interventions to develop a comprehensive and holistic multidimensional activity pacing model that can facilitate tailored discussions about how to improve quality of life through activity pacing. Most intervention studies have relied on practical approaches and applications without in-depth consideration of contemporary theoretical underpinnings. While this approach has its benefits, it may also have limitations. Likewise, very theoretically driven studies often do not include stakeholders’ perceptions, which are important for improving relevance of healthcare interventions. Therefore, this multidimensional model takes advantage of both theories, stakeholders’ perceptions, and practical results of activity pacing interventions, which have shown promise in managing pain and fatigue symptoms among adults with chronic conditions. Moreover, prioritising the use of theories has been suggested in the development of interventions [189]. This review identifies a literature gap on activity pacing and its multidimensionality applied to interventions, thereby underscoring the need for a comprehensive approach that includes the social environment factor.

**Figure 1.**
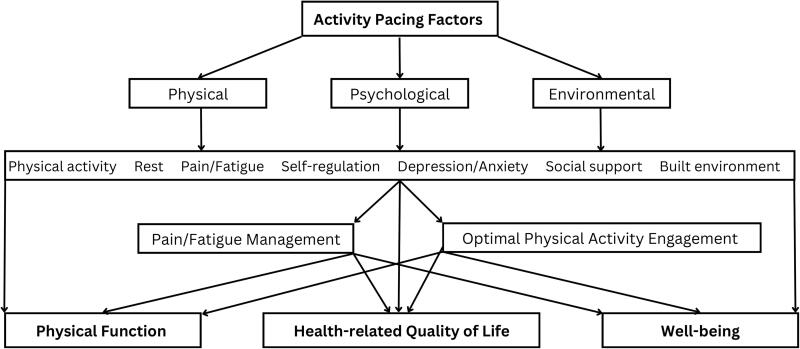
The multidimensional activity pacing concept contributing to achieving a health-related quality of life.

Activity pacing, as a multidimensional concept, aims to be a promising solution for the management of chronic conditions and improvement of HRQoL as depicted in Figure 1. This multidimensional model targets physical, psychological, and environmental factors, enhancing HRQoL, physical function, and well-being as a holistic approach. The various activity pacing factors described in this review illustrate its complexity of this concept, but results are promising. Therefore, a comprehensive and holistic approach to activity pacing is recommended, which may lead to sustained results by helping individuals to optimize their health and well-being and support them in better managing their chronic condition. Moreover, it is important to recognize that activity pacing is a dynamic process that may require adjustments over time to cater to changing needs and circumstances, which is particularly relevant in progressive conditions such as multiple sclerosis. This model accounts for individual differences in activity pacing, including personal preferences, motivations, and beliefs, as well as contextual factors, such as environmental demands and social pressures. The literature highlights the significance of selecting implementation intervention strategies that are tailored to address prevailing barriers [190–192]. Moreover, research shows that individuals value tailored approaches over standardized health care [193,194]. Hence, it is essential to evaluate the current level of functioning, create attainable goals, assess physical and mental health, and provide appropriate levels of motivation and support.

Overall, several studies indicate that activity pacing concept is challenging to be applied in practice [71,72,195]. However, the proposed multidimensional model, drawing on previous empirical and conceptual literature, offers guidance by highlighting the importance of the various dimensions of activity pacing, which potentially might address some of the difficulties. Further research on activity pacing and implementation of this model may reveal stronger benefits, effectively improving pain and fatigue symptoms in adults with chronic conditions while promoting physical activity, ultimately enhancing their HRQoL.

## 6. Conclusion

The present review provides a comprehensive analysis of activity pacing factors and its potential impact on HRQoL of adults with chronic conditions. Health professionals and researchers have leading roles to play in addressing pain and fatigue, and physical inactivity in this population by developing effective adaptations of the activity pacing model introduced in this narrative review. The significant impact of pain and fatigue symptoms on quality of life demands decisive actions from researchers and health professionals to improve the HRQoL of these individuals. The multidimensional approach of the activity pacing model could be a promising solution to the management of pain and fatigue symptoms but also to a sustained physically active lifestyle as this multidimensional model can be used by health professionals and researchers as tool to facilitate discussions about activity pacing in a tailored way. Future research could delve deeper into behavioural change theories within the context of activity pacing to achieve a more comprehensive understanding and optimization.

## Data Availability

Data sharing is not applicable to this article as no new data were created or analyzed in this study.
